# Longitudinal Analysis of Associations Between Dietary Patterns and Incident Suicidal Ideation

**DOI:** 10.3390/nu18172792

**Published:** 2026-08-26

**Authors:** Yulian Kang, Yunzhe Lv, Chenyun Jiang, Qingyue Meng, Ruining Xie, Qin Gao, Yan Liu

**Affiliations:** School of Public Health, Jining Medical University, Jining 272067, China; 18678874555@163.com (Y.K.); 15753831046@163.com (Y.L.); 13863952399@163.com (C.J.); 19558977331@163.com (Q.M.); xieruining1997@mail.jnmc.edu.cn (R.X.); hakunaly@163.com (Y.L.)

**Keywords:** dietary pattern, incident suicidal ideation, mental health

## Abstract

**Background/Objectives**: Diet is a key modifiable determinant of mental health; however, the prospective association between dietary patterns and new-onset suicidal ideation remains poorly elucidated. **Methods**: We enrolled 7480 participants from demolition resettlement communities in Jining, Zoucheng, and Weifang in Shandong Province, China, during 2021–2023. Data were used from two waves (baseline and 1-year follow-up) in this study. Dietary patterns were determined using principal component analysis based on food frequency questionnaire data. Incident suicidal ideation was assessed using the ninth item of the Patient Health Questionnaire-9. Multivariable logistic regression, stratified analysis, and sensitivity analysis were used to examine associations of different dietary patterns with the risk of incident suicidal ideation. **Results**: Among participants, 193 (2.58%) reported incident suicidal ideation. After full multivariable adjustment, the livestock–poultry–seafood dietary pattern was significantly inversely associated with incident suicidal ideation (Q3 vs. Q1; odds ratio = 0.67; 95% confidence interval: 0.45–0.97), whereas no significant association was identified when the pattern score was modelled continuously (OR = 0.92; 95% CI: 0.79–1.06; *p* = 0.239). Stratified analyses revealed stronger inverse associations among men, older adults, overweight individuals, and participants who did not use dietary supplements, but all interaction *p*-values exceeded 0.05, with no statistically confirmed subgroup disparities. After excluding participants with baseline depression, participants with baseline anxiety, and individuals with missing covariates in sensitivity analysis, the inverse association remained statistically stable. **Conclusions**: Our findings suggested a modest inverse association between higher adherence to the livestock–poultry–seafood dietary pattern and new-onset suicidal ideation in this observational cohort.

## 1. Introduction

Suicide is a critical public health issue and a leading cause of death worldwide [1]. According to the World Health Organization, there are over 700,000 annual deaths globally [2], equivalent to approximately one suicide every minute. Suicidal ideation often progresses to suicidal behavior within 1–2 years after onset. The pathophysiology of suicidal ideation is multifactorial, with biological, psychological, and environmental factors jointly implicated in its pathogenesis [3,4,5]. Identifying modifiable factors that may reduce suicidal ideation is therefore an important public health priority. Recently, there has been growing interest in the relationship between dietary factors and suicidal ideation [6,7,8,9,10].

Evidence emerging from nutritional psychiatry reveals consistent correlations between diet and mental and brain health, mediated by an integrated network of biological processes, including inflammation, oxidative stress, epigenetics, mitochondrial dysfunction, gut microbiota and other interrelated metabolic and neuroregulatory pathways [11,12,13]. Broader meta-reviews of lifestyle psychiatry further highlight that diet interacts with physical activity, sleep behaviour and tobacco exposure to affect mental health, with overlapping neurobiological, inflammatory and metabolic mechanisms underlying the associations between diverse lifestyle factors and psychiatric disorders [14]. Multiple studies have confirmed inverse associations between suicidal ideation and individual nutrients, such as dietary fiber, ω-3 polyunsaturated fatty acids, selenium, and niacin [6,15,16,17,18]. Bergmans et al. found that inflammatory factors are important biological markers of suicidal ideation, independent of depressive symptoms [12]. Moreover, anti-inflammatory dietary patterns show inverse associations with lower odds of mental disorders and neurodegenerative diseases [7].

Despite the above evidence, the relationship between dietary patterns and suicidal ideation remains insufficiently elucidated. A nationwide population-based study in Korea found that decreased vegetable intake was associated with an increased risk of suicidal ideation [19]. Based on data from the National Health and Nutrition Examination Survey (NHANES), Li et al. found that specific dietary patterns were associated with suicidal ideation in depressed adults [9]. A systematic review further confirmed that unhealthy dietary behaviors (e.g., high intake of empty-calorie foods) are associated with increased suicidal ideation [20]. However, most of these studies had a cross-sectional design, making it difficult to reveal a temporal relationship.

China is undergoing rapid urbanization, where urban–rural transition and demolition-driven resettlement drastically alter residents’ living conditions, housing modes, and dietary habits, with distinct mental health implications. Nevertheless, few studies have addressed the association of diet with suicidal ideation in relocated populations. Therefore, in this prospective cohort study, we aimed to analyze the association between dietary pattern and incident suicidal ideation among rural and resettlement residents in Shandong Province.

## 2. Materials and Methods

### 2.1. Study Participants

Using a cluster sampling method, we conducted a baseline survey among community residents in Jining City, Zoucheng City, and Weifang City in Shandong Province, China, from April 2021 to July 2023. The three selected cities cover inland and coastal geographic areas of Shandong Province as well as diverse socioeconomic levels and resident lifestyles. Thus, the study area and study population can adequately reflect the overall status of community residents in this region. All participants enrolled in the baseline survey met the following inclusion criteria: (1) age ≥ 18 years; (2) local resident living at the current residential address for no less than 6 months; (3) capable of independently understanding and completing the questionnaire; and (4) voluntary participation with signed informed consent. The exclusion criteria were as follows: (1) individuals with severe somatic diseases; (2) temporary tenants or residents without a fixed and confirmed place of residence; (3) individuals with communication barriers or diagnosed dementia; (4) individuals with missing key baseline information. This study was approved by the Medical Ethics Committee of Jining Medical University (approval number: JNMC-2020-KY-004), and all procedures were performed in accordance with the ethical principles of the Declaration of Helsinki.

A total of 12,480 valid responses were collected at the baseline survey. A follow-up survey was subsequently carried out from July 2023 to July 2024 to dynamically track the incidence of mental disorders and related behavioral outcomes. Uniformly trained investigators conducted standardized face-to-face questionnaire interviews. We applied computer-assisted survey systems with automatic logical verification and skip logic functions throughout the investigation process to ensure the standardization and accuracy of data collection. Daily data quality inspection and random telephone re-verification were performed to minimize measurement bias and guarantee data reliability.

**Figure 1:**
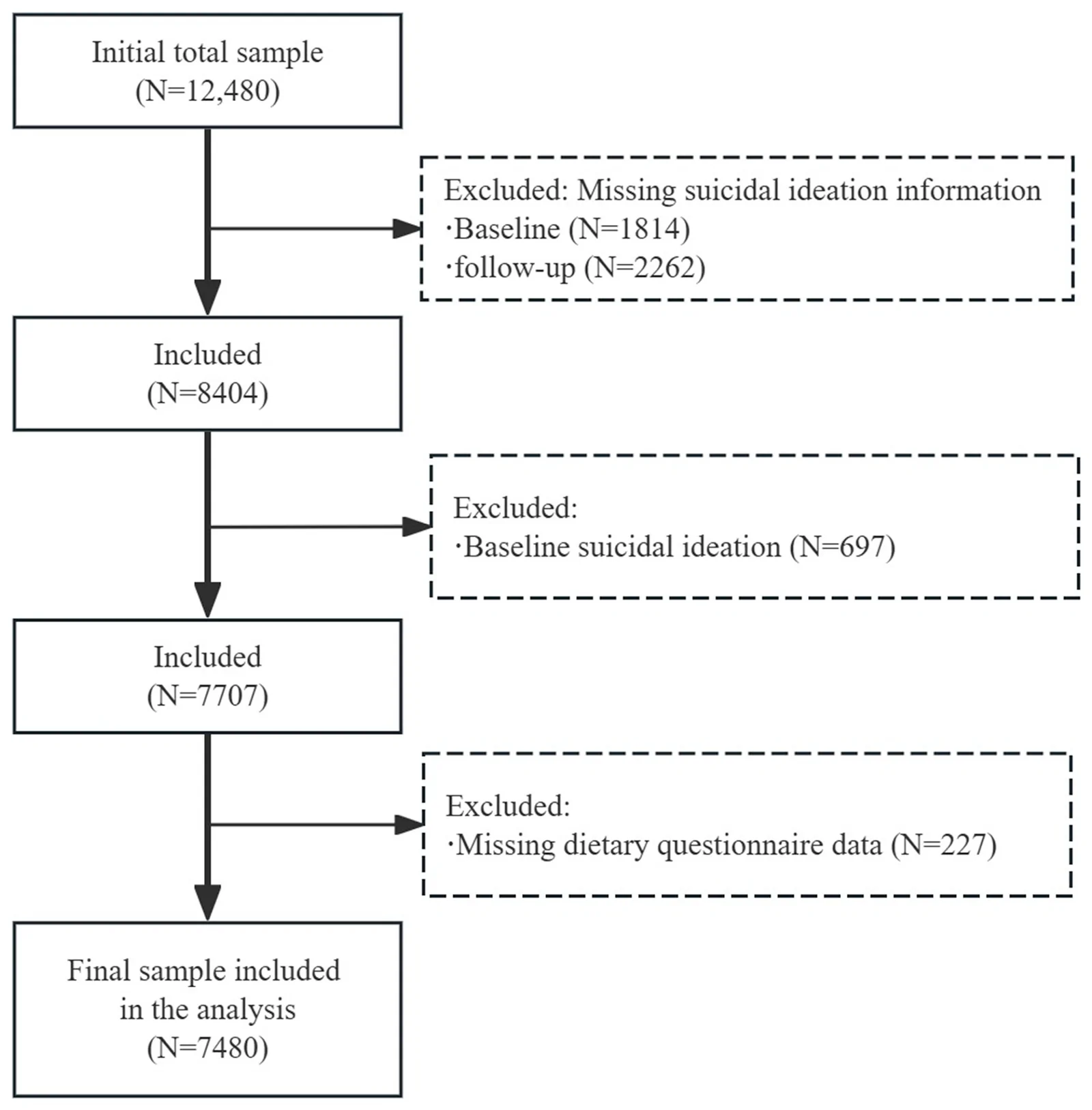
Flow chart of the study. Dashed boxes indicate participants excluded from the study.

After follow-up investigation, strict data screening procedures were implemented to ensure the rigor of statistical analysis. We excluded participants according to the following criteria: missing data on suicidal ideation, individuals with suicidal ideation at baseline, and participants with incomplete dietary questionnaire data. A total of 7480 valid participants were included in the final statistical analysis. The detailed study design procedure and participant screening flow chart are illustrated in Figure 1.

To evaluate potential selection bias introduced by participant exclusion, we compared baseline sociodemographic, lifestyle, and health characteristics between participants retained in the final analytical sample and those excluded due to missing suicidal ideation data. Minor differences in several baseline indicators were observed, and the detailed comparative results are presented in Table S1.

### 2.2. Dietary Assessment

We used a food frequency questionnaire to assess the dietary intake of participants over the past year. The questionnaire covered 22 common food categories, including grains, vegetables, fruit, meat, eggs, dairy products, and nuts. Dietary frequency was scored on a 5-point scale: 1 (rarely/never), 2 (occasional, <1/month), 3 (≥1/month), 4 (≥1/week), and 5 (almost daily). Scores quantitatively reflected the long-term dietary habits of study participants.

We used principal component analysis (PCA) with varimax rotation to determine participants’ dietary patterns [21]. Suitability of the data for PCA was assessed using the Kaiser–Meyer–Olkin test (threshold > 0.6) and Bartlett’s sphericity test (significance level *p* < 0.001). To objectively determine the optimal number of retained dietary components, we adopted four mutually complementary statistical and interpretive criteria simultaneously: (1) eigenvalue cutoff ≥ 1.0; (2) clear inflection point on the scree plot (Figure S1); (3) cumulative explained variance reaching 60.9% after extracting six factors; and (4) clear, interpretable food group loadings for each component. After cross-verification of the four standards, six interpretable dietary patterns were finally retained. Food groups with absolute factor loadings ≥ 0.40 were considered core contributors to each pattern. Individual dietary pattern scores were calculated as the weighted sum of standardized food intake frequencies, using factor loadings as weights. Higher scores indicated closer conformity to the corresponding dietary patterns.

### 2.3. Assessment of Incident Suicidal Ideation

Suicidal ideation was screened using participants’ responses to item 9 of the 9-item Patient Health Questionnaire (PHQ-9) [9,22,23], which is as follows: “Have you been troubled by thoughts of feeling like you would be better off dead, or that you’re going to harm yourself, in the past 2 weeks?” In this study, suicidal ideation was defined as a score of 1–3 on item 9 of the PHQ-9, corresponding to the response options “several days,” “more than half the days,” or “nearly every day.” A score of 0 (“not at all”) was classified as no suicidal ideation. This single PHQ-9 item serves only as a population screening indicator rather than a formal clinical diagnostic tool for suicidal ideation.

### 2.4. Assessment of Depressive Symptoms

Depressive symptoms were measured using the PHQ-9, which was developed in accordance with diagnostic criteria of the Diagnostic and Statistical Manual of Mental Disorders, Fourth Edition [24]. The PHQ-9 scale is used to retrospectively evaluate depressive manifestations over the preceding 2 weeks using nine items, rated on a 4-point Likert scale ranging from 0 (never) to 3 (nearly every day), yielding a total score from 0 to 27 [25]. Standard cutoff values of 5, 10 and 15 were adopted to categorize depressive symptoms into mild, moderate, and severe levels, respectively [26].

### 2.5. Assessment of Anxiety Symptoms

Anxiety symptoms were assessed using the validated 7-item Generalized Anxiety Disorder-7 (GAD-7) scale developed by Spitzer et al. in 2006 [27]. Each item assesses anxiety symptoms over the previous 2 weeks, rated on a 4-point Likert scale ranging from 0 (not at all) to 3 (nearly every day). Total scores range from 0 to 21, with higher scores indicating more severe anxiety. The GAD-7 exhibits good sensitivity (81.1%) and specificity (84.5%) and has been verified as having favorable reliability and validity among Chinese populations [28].

### 2.6. Covariates

In this study, various potential confounding variables including sociodemographic factors, behavioral characteristics, and anthropometric measurements were selected as covariates. Sociodemographic variables included sex, age, education level, residential location, and marital status. Educational level was classified into three categories: ≤6 years, 7–12 years, and >12 years. Residential location was categorized as rural and new-urban. Marital status was recoded into three categories: married (including first marriage, remarriage, and marital separation), unmarried (including cohabiting, dating, and single individuals), and other (divorced or widowed).

Behavioral factors included smoking status, alcohol drinking status, tea drinking status, and dietary supplement use. Smoking status was grouped as “never smoked” (no smoking in the previous 12 months) and “ever smoked” (any active smoking in the previous 12 months, including heavy, regular, occasional, and social smoking). Alcohol consumption status was grouped into “non-drinker” (no alcohol intake in the previous 12 months) and “drinker” (any alcohol consumption in the previous 12 months, including heavy, moderate, light, and social drinking). Tea consumption status was categorized into “non-tea drinker” (no tea intake in the previous 12 months) and “tea drinker” (any tea consumption in the previous 12 months, including occasional and regular tea drinking). Dietary supplement use was grouped into “yes” (regular or occasional dietary supplement intake in the previous 12 months) and “no” (no dietary supplement intake in the previous 12 months).

Height and weight were measured by trained investigators using standard protocols. Height was measured to the nearest 0.1 cm using a calibrated stadiometer, with participants standing barefoot and in an upright position. Weight was measured to the nearest 0.1 kg using a calibrated digital scale, with participants wearing light indoor clothing and no shoes. Body mass index (BMI) was calculated based on height and weight (BMI = weight (kg)/height (m)^2^) and classified into four categories: underweight (<18.5 kg/m^2^), normal (18.5–23.9 kg/m^2^), overweight (24.0–27.9 kg/m^2^), and obese (≥28.0 kg/m^2^).

### 2.7. Statistical Methods

Categorical variables are expressed as frequency and percentage; normally distributed continuous variables are presented as mean ± standard deviation. Group differences were analyzed using t-tests and chi-squared tests. Dietary pattern scores were categorized into tertiles. Logistic regression was used to examine their associations with new-onset suicidal ideation, and odds ratios (ORs) and 95% confidence intervals (CIs) were reported. The crude model included no covariates. Model 1 was adjusted for sex, age and BMI. On the basis of Model 1, Model 2 was further adjusted for educational level, residential location, marital status, smoking, alcohol consumption, tea consumption, and dietary supplement use. To account for selection bias caused by missing outcome data, we performed inverse probability weighting (IPW). Baseline variables were used to calculate stabilized weights for weighted logistic regression to re-examine the association between dietary patterns and incident suicidal ideation.

To explore potential heterogeneity, we performed stratified analyses according to age, sex, BMI categories, marital status, educational level, residence status, smoking status, alcohol-drinking status, tea-drinking status, and dietary supplement use. Interaction effects were assessed by adding a multiplicative interaction term between dietary pattern scores and the corresponding stratification variable.

We performed exploratory parallel mediation analysis using the PROCESS macro (version 3.4.1, Model 4) implemented in R. Covariates incorporated into the adjusted analytical framework encompassed sex, age, BMI, education level, residential location, marital status, smoking status, alcohol intake, tea consumption, and dietary supplement use. In the mediation model, dietary patterns were specified as the independent variable, anxiety and depressive symptoms served as two parallel mediating variables, and suicidal ideation was treated as a binary outcome variable. Bias-corrected bootstrapping with 1000 replicated samples was used to quantify indirect mediating effects.

Sensitivity analyses were conducted to evaluate robustness of the primary findings. Three sensitivity scenarios were implemented: (1) excluding participants with baseline depression symptoms, defined as a total PHQ-9 score ≥ 5 (*n* = 158); (2) excluding participants with baseline anxiety symptoms, defined as a GAD score ≥ 10 (*n* = 20); and (3) excluding participants with missing values for basic characteristics on the questionnaire (*n* = 224).

All statistical tests were two-sided, and *p* < 0.05 was considered statistically significant. Analyses were performed using R software version 4.5.2 (The R Project for Statistical Computing, Vienna, Austria) and EmpowerStats software (version 2.0, X&Y Solutions, Inc., Boston, MA, USA, http://www.empowerstats.com).

## 3. Results

### 3.1. Basic Characteristics of Study Population

Among 7480 participants, most were women (58.68%), middle-aged or older (mean age = 65 years), married (82.67%), and had a low educational level (85.86%). A total of 193 participants (2.58%) developed incident suicidal ideation during the 1-year follow-up. Both residential location and dietary supplement use differed significantly according to incident suicidal ideation (both *p* < 0.001). New-urban residents had a higher proportion of new-onset suicidal ideation (1.71%) than rural residents (0.87%). Participants who used dietary supplements had a lower prevalence of incident suicidal ideation (0.23%) than non-users (2.34%) (Table 1).

### 3.2. Identified Dietary Patterns

**Figure 2:**
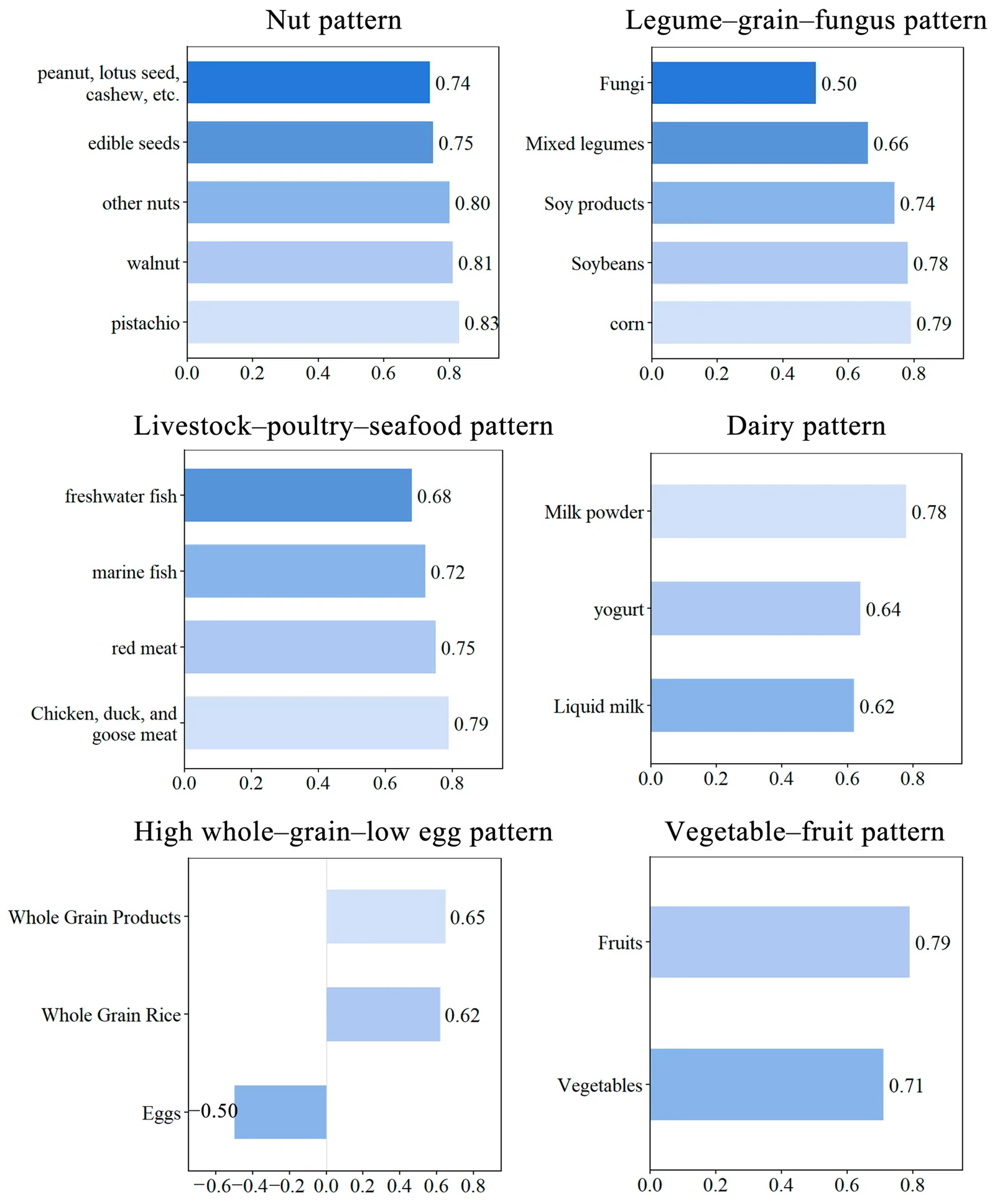
Factor loadings of food items for the six extracted dietary patterns derived from principal component analysis. Within each dietary–pattern subplot, darker–colored bars correspond to lower factor–loading values, and lighter–colored bars represent higher factor–loading values.

Through analysis of the scree plot (Figure S1), six dietary patterns were identified with eigenvalues above 1.0 and factor loadings above 0.40. These six major patterns collectively accounted for 60.9% of the total variance in food intake frequency. The key food items and their respective factor loadings for each pattern were as follows: (1) the nut pattern, characterized by regular consumption of pistachios, walnuts, edible seeds, peanuts, and other nuts; (2) the legume–grain–fungus pattern, defined by intake of corn, soybeans, soy products, mixed legumes, and fungi; (3) the livestock–poultry–seafood pattern, including regular consumption of poultry (chicken, duck, and goose meat), red meat, marine fish, and freshwater fish; (4) the dairy pattern, comprising milk powder, yogurt, and liquid milk intake; (5) the high-whole-grain–low egg pattern, marked by positive loadings for whole-grain products and whole-grain rice, alongside a negative loading for eggs; and (6) the vegetable–fruit pattern, dominated by consumption of fruits and vegetables (Figure 2).

### 3.3. Correlation Between Dietary Patterns and Incident Suicidal Ideation

Table 2 presents the associations between the above six dietary patterns and the risk of incident suicidal ideation. No significant associations were observed for the nut, legume–grain–fungus, and dairy patterns across all models. For the livestock–poultry–seafood pattern, the highest tertile showed a significantly lower risk of incident suicidal ideation than the lowest tertile in the crude model (OR = 0.63; 95% CI: 0.44 to 0.91), Model 1 (OR = 0.67; 95% CI: 0.44 to 0.92), and the fully adjusted Model 2 (OR = 0.67; 95% CI: 0.45 to 0.97), with a significant decreasing trend across tertiles (*p* for trend = 0.016, 0.020, and 0.045, respectively). For the high-whole-grain–low-egg pattern, both the crude model and Model 1 showed a significantly higher risk of incident suicidal ideation in Q3 than in Q1 (crude: OR = 1.51 and 95% CI: 1.07 to 2.12; Model 1: OR = 1.56 and 95% CI: 1.10 to 2.20), with a significant positive trend (*p* for trend = 0.013 and 0.008, respectively). However, these associations were attenuated and no longer significant in the fully adjusted Model 2 (OR = 1.17; 95% CI: 0.81 to 1.69; *p* for trend = 0.311). For the vegetable–fruit pattern, a significant inverse trend across tertiles was observed in the crude model and Model 1 (*p* for trend = 0.019 and 0.023, respectively), although this trend did not persist after adjustment for all covariates.

IPW analysis was performed to test the robustness of findings against potential selection bias from missing outcome data. The IPW-weighted results were largely consistent with the primary unweighted analyses (Table S2). For the livestock–poultry–seafood pattern, the weighted OR for Q3 versus Q1 was 0.68 (95% CI: 0.46–1.00), closely matching the fully adjusted unweighted estimate. No meaningful shifts in associations were detected for the remaining dietary patterns.

### 3.4. Subgroup Analyses and Sensitivity Analyses

As shown in Table 3, subgroup analyses revealed that, among participants aged ≥60 years, men, individuals with a BMI ≥ 28 kg/m^2^, and dietary supplement users, the highest tertile of the livestock–poultry–seafood pattern was inversely associated with incident suicidal ideation relative to the lowest tertile. A significant linear decreasing trend was also observed across tertiles in these subgroups. Notably, no significant interactions were identified between the livestock–poultry–seafood pattern and any stratifying factor. Results of subgroup analyses for the remaining dietary patterns are presented in Table S3 and were consistent with the main analyses.

Sensitivity analyses via multiple exclusion criteria yielded consistent results; across all sensitivity models, Q3 was significantly associated with lower odds of suicidal ideation than Q1 (OR = 0.63, *p* < 0.05), confirming the reliability of the main findings (Figure S2).

### 3.5. Mediation Analysis

Table S4 shows the analysis results for the livestock–poultry–seafood dietary pattern and baseline depression and anxiety symptoms (potential mediators). After adjustment for confounding factors, participants in the Q2 tertile of the livestock–poultry–seafood dietary pattern exhibited significantly lower PHQ-9 scores (β = −0.12, 95% CI: −0.18 to −0.05, *p* < 0.001) and GAD scores (β = −0.18, 95% CI: −0.26 to −0.09, *p* < 0.001) relative to the Q1 reference group. For the Q3 tertile, a significant inverse association was still observed between this dietary pattern and PHQ-9 scores (β = −0.08, 95% CI: −0.14 to −0.02, *p* = 0.012), whereas no significant correlation was detected for GAD scores (β = −0.06, 95% CI: −0.15 to 0.02, *p* = 0.129).

**Figure 3:**
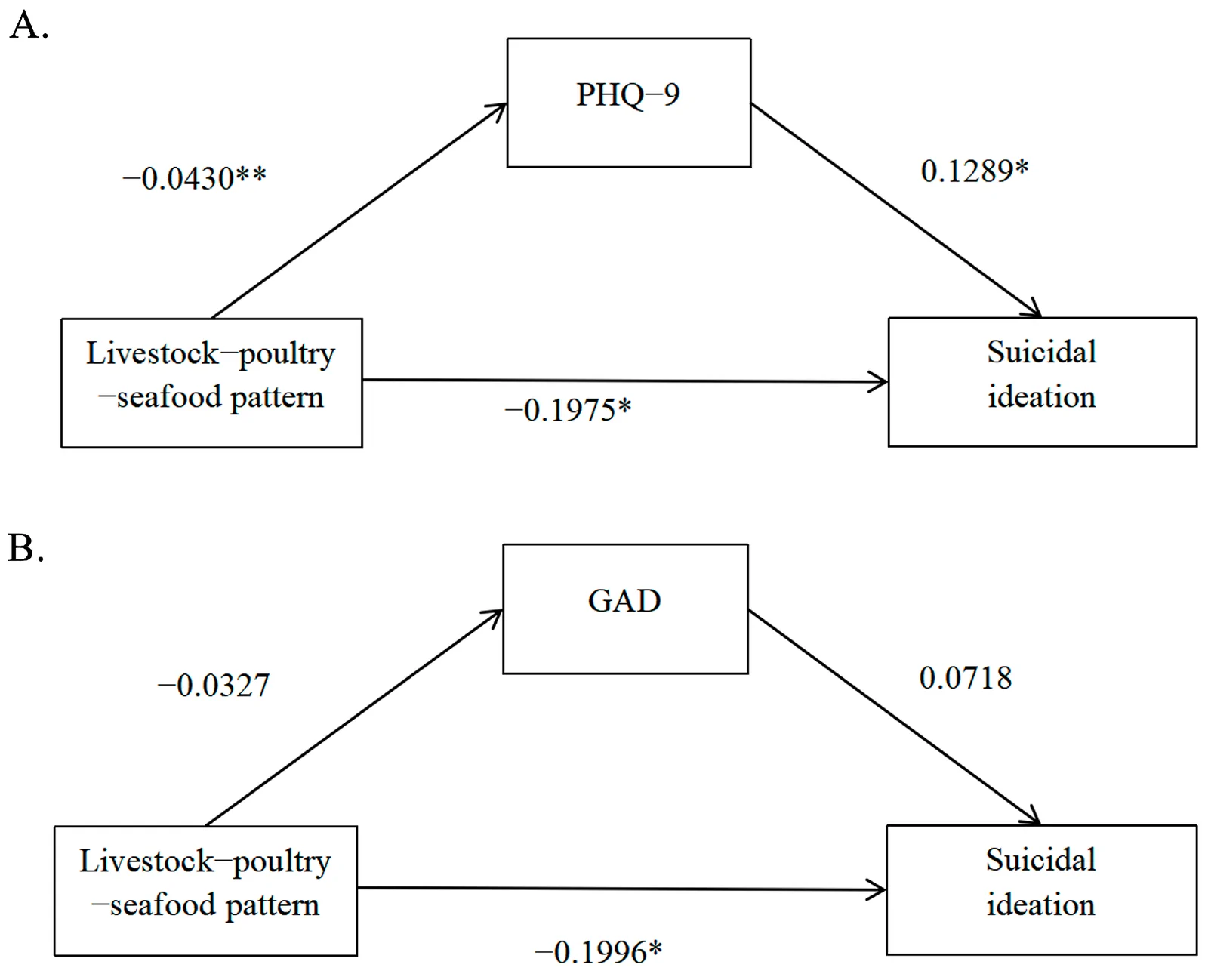
Mediation analysis of depressive and anxiety symptoms in the association between the livestock–poultry–seafood dietary pattern and incident suicidal ideation. (**A**) Mediation pathway of PHQ–9 depressive symptoms; (**B**) Mediation pathway of GAD–based anxiety symptoms. * *p* < 0.05, ** *p* < 0.01.

Next, we performed mediation analysis to assess whether the association between dietary pattern and suicidal ideation was mediated through baseline depression and anxiety symptoms (Figure 3). After controlling for PHQ-9 scores and all the aforementioned covariates, the livestock–poultry–seafood dietary pattern showed a significant direct inverse association with the odds of suicidal ideation: β = −0.1874 (OR = 0.829, 95% CI: 0.692 to 0.994, *p* = 0.042). However, the indirect effect of this dietary pattern on suicidal ideation via PHQ-9 scores was −0.0050 (bootstrap 95% CI: −0.0129 to 0.0005), indicating no significant mediating role of depressive symptoms. Following adjustment for GAD scores and all covariates, the dietary pattern demonstrated a significant direct inverse association with the odds of suicidal ideation (direct β = −0.1890, OR = 0.828, 95% CI: 0.691 to 0.992, *p* = 0.0408). The indirect effect through GAD was −0.0022 (bootstrap 95% CI: −0.0068 to 0.0008).

## 4. Discussion

In the current study, using data from a large population-based cohort of residents in demolition resettlement communities and rural areas of China, we provided evidence of a longitudinal relationship between six dietary patterns and suicidal ideation. Our analysis showed a significant association between higher adherence to the livestock–poultry–seafood pattern and incident suicidal ideation when participants were split into tertiles, whereas no statistically meaningful correlation was observed for continuous standardized pattern scores. Stratified analyses pointed to more pronounced inverse correlations among men, older adults aged 60 years and above, overweight individuals (24 ≤ BMI < 28 kg/m^2^), and non-users of dietary supplements; however, all interaction terms were non-significant, offering no statistical proof of genuine subgroup effect modification. Multiple sensitivity analyses further confirmed the robustness of this core inverse association after excluding participants with baseline depressive symptoms, those with baseline anxiety symptoms, and individuals with missing covariate information. Of note, crude associations for the vegetable–fruit and high-whole-grain–low egg patterns attenuated to non-significance after full adjustment, suggesting confounding and no consistent independent inverse associations.

Recent years have witnessed a rapid expansion in suicide-related studies; however, no universally agreed definition or classification criteria for suicidal behavior have been established so far [29]. Based on the ideation-to-action theoretical model, suicidal ideation acts as the precursor of suicidal behaviors, whereas suicide capability plays a decisive role in the transformation from ideation to actual action [30]. In this context, research focusing on the association between dietary patterns and suicidal ideation remains insufficient [9,31]. A cross-sectional study conducted among 9002 Korean adults found no overall association between general dietary patterns and suicidal ideation [31]. The Healthy Eating Index-2015, Dietary Inflammatory Index, Comprehensive Dietary Antioxidant Index, Oxidative Balance Score, and Dietary Index for Gut Microbiota (DI-GM) are widely used to assess dietary patterns associated with inflammation, antioxidant capacity, and gut microbiota diversity. Analysis of NHANES data demonstrated that DI-GM was the only one among the five indices that was independently associated with a lower risk of comorbid depression and suicidal ideation, particularly in metabolically vulnerable groups. This supports the hypothesis that diet-mediated gut microbiota diversity may influence the progression from depression to suicidal ideation via the gut–brain axis.

The inverse association of the livestock–poultry–seafood dietary pattern against suicidal ideation can be interpreted from the perspective of nutritional psychiatry, which involves multiple interconnected mechanisms. This dietary pattern is rich in key nutrients such as high-quality animal protein, ω-3 polyunsaturated fatty acids (PUFAs, with EPA (eicosapentaenoic acid) and DHA (docosahexaenoic acid) as the main active components), selenium, and zinc, which may jointly contribute to neurochemical homeostasis and anti-inflammatory defense.

First, high-quality dietary protein provides essential amino acids including tryptophan and tyrosine, which are the primary substrates for synthesizing serotonin and dopamine. Deficits in neurotransmitter signaling are well-established biological contributors to suicidal behaviors [32]. Omics-based investigations have revealed pervasive dysregulation of the serotonergic system and other neurotransmitter pathways in the brain of suicide decedents. Such neural abnormalities, together with impaired glutamatergic/gamma–aminobutyric acid–ergic transmission, glial cell dysfunction and sustained neuroinflammation, constitute the complex pathological network of suicidal ideation [33].

The anti-inflammatory properties of ω-3 PUFAs may partly explain their potential inverse association with suicidal ideation. Epidemiological evidence demonstrates that increased ω-3 PUFA intake is correlated with a lower prevalence of suicidal ideation, an effect largely mediated by the suppression of chronic inflammation and downregulation of pro-inflammatory molecules such as *C*-reactive protein (CRP) [15]. Nonetheless, the precise dose–response thresholds and underlying molecular cascades need systematic verification. Accumulating evidence also indicates that inflammatory biomarkers (CRP, interleukin-6) are reliable indicators of suicidal ideation, particularly in elderly people [34].

Additionally, a recent large-sample study based on the NHANES clearly indicated that dietary selenium intake is significantly negatively correlated with suicidal ideation. Each one-unit increase in selenium intake corresponded to a 41% reduction in the risk of suicidal ideation (OR = 0.59; 95% CI = 0.41 to 0.85) [18]. As a vital constituent of glutathione peroxidase, selenium possesses powerful antioxidant activity and protects neurons from oxidative damage, which may account for its neuroprotective role relative to suicidal ideation. Collectively, the above evidence indicates that adherence to the livestock–poultry–seafood dietary pattern correlates with a lower likelihood of suicidal ideation via coordinated regulation of the neurotransmitter–inflammation–oxidative stress axis.

Our subgroup analyses revealed that the inverse association between the livestock–poultry–seafood dietary pattern and incident suicidal ideation was more pronounced among men, adults aged ≥60 years, overweight individuals (24 ≤ BMI < 28 kg/m^2^) and non-users of dietary supplements. National suicide statistics across China (2002–2021) reveal an age-dependent upward trend in suicide rates, alongside a strong male predominance in suicide risk observed after 2005 [35]. As such, greater compliance with the aforementioned dietary pattern might confer a more pronounced risk-reduction benefit for new-onset suicidal ideation within these high-risk groups. Numerous epidemiological studies have identified a positive relationship between BMI and suicidality in adults as well as adolescents [36,37,38]. Multiple psychosocial and biological mechanisms can explain the higher baseline risk of suicidal ideation among individuals with elevated BMI. First, weight-related social stigma, negative body self-perception, and repeated failure in terms of weight management contribute to persistent psychological distress, which is a critical trigger for suicidal thoughts [39,40]. Moreover, physical inactivity, a prevalent lifestyle characteristic among overweight and obese populations, exacerbates both metabolic dysfunction and mood disorders, further compounding suicide risk [20]. Biologically, obesity-related metabolic disorders such as diabetes and metabolic syndrome alter the insulin and serotonin balance alongside glucose and lipid dysregulation, which together impair emotional control and raise the suicide risk [17,41,42]. Notably, formal interaction tests failed to reach statistical significance. Accordingly, we cannot confirm genuine effect modification across subgroups. These stratified associations are merely exploratory and require further independent cohort research for validation.

Accumulating evidence indicates that a variety of mental disorders may interfere with the relationship between dietary patterns and suicidal ideation. Among these, anxiety and depression are particularly noteworthy as they may alter individuals’ dietary preferences and behaviors. Consistent with established nutritional psychiatry frameworks, dietary patterns are cross-sectionally associated with depressive and anxious symptoms, and mood disturbances are known predictors of suicidal ideation [43,44]. Coupled with the temporal sequence of our cohort data, we treated baseline depression and anxiety as candidate mediators for exploratory mechanistic inference. Although the livestock–poultry–seafood pattern was cross-sectionally associated with lower baseline depression and anxiety levels, no significant indirect pathways through PHQ-9 or GAD scores were detected. A significant direct effect persisted after accounting for emotional symptoms. Furthermore, we conducted a sensitivity analysis by excluding participants who presented with anxiety or depressive symptoms at baseline. The results demonstrated that the core inverse association between the livestock–poultry–seafood dietary pattern and incident suicidal ideation remained stable after exclusion. Taken together, these results indicate that the beneficial influence of the livestock–poultry–seafood dietary pattern on incident suicidal ideation is independent of baseline depressive and anxiety symptoms. As emphasized by Spinoni et al. [45], psychological symptoms represent dynamic, multifaceted constructs with complex longitudinal and bidirectional relationships to health outcomes; relying solely on single-time baseline measurements of mood may fail to capture evolving affective states across follow-up and thus limit the detection of mediating pathways. Beyond classic neurotransmitter and broad anti-inflammatory frameworks, nutritional psychiatry research points toward alternative plausible biological routes, among which microbiota-gut–brain interactions deserve particular attention [46]. Notably, gut–brain axis pathways are not restricted to the regulation of depressive and anxious mood. While gut microbiota is well-established to modulate emotional processing, gut-derived metabolic and immune signals may also alter stress resilience, neuroinflammatory status and impulse-related brain circuits partly independent of clinically manifest mood disturbances.

This study has several limitations. First, several measurement limitations exist in our dietary assessment. The food frequency questionnaire only collects food consumption frequency rather than precise intake quantities, which may introduce measurement bias to dietary exposure data. Additionally, dietary patterns extracted via principal component analysis are inherently sample-specific; the combination and structure of identified food factors cannot be directly replicated in cohorts with distinct food cultures, socioeconomic backgrounds, or regional dietary habits, limiting cross-population generalizability of our dietary pattern findings. Second, our measurement of incident suicidal ideation relies solely on item 9 of the PHQ-9 screening scale, which carries important inherent limitations. This single two-week retrospective item cannot distinguish passive death wishes from deliberate self-harm ideation and lacks the comprehensive coverage of standardized suicide-specific assessment tools. Accordingly, our outcome only represents positive screening responses rather than formally diagnosed clinical suicidal ideation, which may attenuate direct comparability with studies adopting dedicated suicide evaluation instruments. Third, despite adjustment for multiple demographic and behavioral covariates, several established predictors of suicidal ideation were not captured in our dataset, such as psychiatric history, psychotropic medication use, prior suicide attempts, chronic illnesses, sleep quality, social support, stressful life events, and socioeconomic hardship. Residual confounding from these unmeasured factors may contribute to the observed association and cannot be excluded. Fourth, selection bias may arise from the application of strict inclusion and exclusion criteria, which could restrict the representativeness of the analytical sample. We performed inverse probability weighting (IPW) as a sensitivity analysis to mitigate such selection bias by weighting participants according to their probability of being included in the final analytic cohort. Fifth, the study population mainly included residents in resettlement communities and rural areas, which may limit the generalizability of our findings to other populations, such as urban residents or those with different socioeconomic backgrounds. Despite these limitations, a key strength of this study is its prospective design, which allowed us to establish the temporal sequence of the association between dietary patterns and incident suicidal ideation. Stratified and sensitivity analyses further confirmed the stability of the results.

The above results suggest that adequate intake of high-quality animal protein may correlate with better mental health among middle-aged and older people in the community. In suicide prevention work, against a background of urbanization, personalized dietary guidance strategies can be formulated in line with the characteristics of different residential locations. For high-risk groups such as new urban residents, further research may focus on health education around high-quality animal protein intake. At the same time, the living environment and social support system in resettlement communities after demolition should be optimized. Further research may explore whether coordinated improvements in dietary habits and social environments could correlate with lower suicidal ideation risk among community residents. Given that individuals with mental disorders often have poor diet quality, systematic implementation of nutrition and lifestyle interventions in mental health care is of great importance in suicide prevention [47,48]. Combined with the key role of suicide capability in the ideation-to-action transition [30], this study provides new insight and evidence for the early prevention of suicide in the field of public health.

## 5. Conclusions

In this prospective observational cohort study, higher adherence to the livestock–poultry–seafood dietary pattern was modestly associated with lower odds of incident suicidal ideation after full covariate adjustment. Further prospective and interventional studies are needed to clarify the biological mechanisms underlying this association.

## Figures and Tables

**Table 1:** Distribution of basic demographic characteristics of the study subjects.

Characteristics	Total Population	Incident Suicidal Ideation		*p*
		Yes	No	
N (%)	7480 (100)	193 (2.58)	7287 (97.42)	
Age (years)	65.18 ± 14.23	66.20 ± 12.03	65.15 ± 14.28	0.311
Sex (%)				0.631
Female	3091 (41.32)	83 (43.01)	3008 (41.27)	
Male	4389 (58.68)	110 (56.99)	4279 (58.73)	
BMI (kg/m^2^)	24.64 ± 4.43	24.09 ± 3.80	24.68 ± 4.42	0.083
Marital status (%)				0.387
Married	6184 (82.67)	166 (86.01)	6018 (82.59)	
Unmarried	216 (2.89)	6 (3.11)	210 (2.88)	
Other	1068 (14.28)	21 (10.88)	1047 (14.37)	
Missing	12 (0.16)	0 (0.00)	12 (0.16)	
Education (%)				0.186
≤6 years	6422 (85.86)	168 (87.05)	6254 (85.82)	
>7 years	979 (13.09)	22 (11.40)	957 (13.13)	
Missing	79 (1.06)	3 (1.55)	76 (1.04)	
Residence status (%)				<0.001
Rural	3670 (49.06)	65 (33.68)	3605 (49.47)	
New-Urban	3718 (49.70)	127 (65.80)	3591 (49.28)	
Urban	55 (0.94)	1 (0.52)	54 (0.74)	
Missing	37 (0.49)	0 (0.00)	37 (0.51)	
Smoking status (%)				0.089
Never smoked	5946 (79.49)	144 (74.61)	5802 (79.62)	
Ever smoked	1534 (20.51)	49 (25.39)	1485 (20.38)	
Alcohol-drinking status (%)				0.725
Non-drinker	5892 (78.77)	154 (79.79)	5738 (78.74)	
Drinker	1588 (21.23)	39 (20.21)	1549 (21.26)	
Tea-drinking status (%)				0.723
Non-tea drinker	3275 (43.78)	82 (42.49)	3193 (43.82)	
Tea drinker	4175 (55.82)	110 (56.99)	4065 (55.78)	
missing	30 (0.40)	1 (0.52)	29 (0.40)	
Dietary supplements use (%)				<0.001
Yes	1485 (19.85)	17 (8.81)	1468 (20.15)	
No	5963 (79.72)	175 (90.67)	5788 (79.43)	
Missing	32 (0.43)	1 (0.52)	31 (0.42)	
PHQ-9 (%)				0.067
<5	7322 (97.89)	185 (95.85)	7137 (97.94)	
≥5	158 (2.11)	8 (4.15)	150 (2.06)	
GAD (%)				0.408
<10	7459 (99.72)	192 (99.48)	7267 (99.73)	
≥10	20 (0.27)	1 (0.52)	19 (0.26)	

**Table 2:** Association between dietary patterns and risk of new-onset suicidal ideation.

	Case/*N*	Crude Model	Model 1	Model 2
Nut pattern				
Q1 (−2.78, −0.77)	57/2493	1.00 (Ref.)	1.00 (Ref.)	1.00 (Ref.)
Q2 (−0.77, −0.36)	68/2493	1.18 (0.83, 1.70)	1.23 (0.85, 1.76)	1.16 (0.80, 1.68)
Q3 (−0.36, 9.79)	68/2494	1.28 (0.89, 1.82)	1.35 (0.94, 1.94)	1.15 (0.79, 1.68)
*p* for trend	—	0.182	0.108	0.471
Per SD increase	—	0.99 (0.86, 1.14)	1.01 (0.87, 1.17)	0.95 (0.82, 1.10)
*p*	—	0.872	0.926	0.505
Legume–grain–fungus pattern				
Q1 (−3.79, −1.05)	62/2493	1.00 (Ref.)	1.00 (Ref.)	1.00 (Ref.)
Q2 (−1.05, −0.46)	59/2493	0.93 (0.65, 1.34)	0.94 (0.65, 1.35)	0.91 (0.63, 1.31)
Q3 (−0.46, 3.16)	72/2494	1.18 (0.84, 1.67)	1.20 (0.85, 1.70)	1.03 (0.72, 1.47)
*p* for trend	—	0.327	0.282	0.850
Per SD increase	—	1.02 (0.89, 1.18)	1.03 (0.89, 1.19)	0.97 (0.84, 1.12)
*p*	—	0.742	0.669	0.703
Livestock–poultry–seafood pattern				
Q1 (−3.17, −1.15)	75/2493	1.00 (Ref.)	1.00 (Ref.)	1.00 (Ref.)
Q2 (−1.15, −0.44)	70/2493	0.93 (0.67, 1.30)	0.93 (0.67, 1.30)	1.05 (0.75, 1.47)
Q3 (−0.44, 6.34)	48/2494	0.63 (0.44, 0.91)	0.67 (0.44, 0.92)	0.67 (0.45, 0.97)
*p* for trend	—	0.016	0.020	0.045
Per SD increase	—	0.89 (0.78, 1.02)	0.89 (0.78, 1.04)	0.92 (0.79, 1.06)
*p*	—	0.096	0.124	0.239
Dairy pattern				
Q1 (−2.69, −1.07)	68/2493	1.00 (Ref.)	1.00 (Ref.)	1.00 (Ref.)
Q2 (−1.07, −0.55)	71/2493	1.05 (0.74, 1.47)	1.05 (0.75, 1.47)	1.12 (0.79, 1.58)
Q3 (−0.55, 3.62)	54/2494	0.83 (0.58, 1.19)	0.84 (0.58, 1.20)	0.88 (0.61, 1.27)
*p* for trend	—	0.325	0.349	0.505
Per SD increase	—	0.90 (0.78, 1.04)	0.90 (0.78, 1.04)	0.92 (0.80, 1.07)
*p*	—	0.145	0.161	0.281
High-whole-grain–low egg pattern				
Q1 (−3.63, −0.97)	56/2492	1.00 (Ref.)	1.00 (Ref.)	1.00 (Ref.)
Q2 (−0.97, −0.44)	52/2494	0.89 (0.61, 1.31)	0.91 (0.62, 1.34)	0.83 (0.56, 1.23)
Q3 (−0.44, 4.52)	85/2494	1.51 (1.07, 2.12)	1.56 (1.10, 2.20)	1.17 (0.81, 1.69)
*p* for trend	—	0.013	0.008	0.311
Per SD increase	—	1.18 (1.03, 1.36)	1.20 (1.04, 1.38)	1.07 (0.91, 1.24)
*p*	—	0.021	0.013	0.414
Vegetable–fruit pattern				
Q1 (−8.43, −0.91)	71/2493	1.00 (Ref.)	1.00 (Ref.)	1.00 (Ref.)
Q2 (−0.91, −0.26)	76/2493	1.01 (0.72, 1.40)	1.02 (0.73, 1.42)	1.11 (0.79, 1.55)
Q3 (−0.26, 1.63)	46/2494	0.63 (0.44, 0.92)	0.64 (0.44, 0.93)	0.74 (0.50, 1.08)
*p* for trend	—	0.019	0.023	0.159
Per SD increase	—	0.99 (0.86, 1.13)	0.99 (0.86, 1.14)	1.04 (0.89, 1.21)
*p*	—	0.841	0.916	0.637

The crude model included no covariates. Model 1 was adjusted for sex, age and BMI. On the basis of Model 1, Model 2 was further adjusted for educational level, residence type, marital status, smoking, alcohol-drinking, tea-drinking status and dietary supplements use.

**Table 3:** Stratified analysis of the livestock–poultry–seafood dietary pattern and new-onset suicidal ideation.

Characteristic	Q1	Q2	Q3	*p* for Trend	*p* for Interaction
Age (years)					0.537
Age < 60	Ref.	1.29 (0.59, 2.82)	0.98 (0.46, 2.10)	0.883	
Age ≥ 60	Ref.	0.97 (0.67, 1.41)	0.55 (0.35, 0.86)	0.035	
Sex (%)					0.172
Male	Ref.	0.96 (0.63, 1.47)	0.48 (0.28, 0.82)	0.019	
Female	Ref.	1.13 (0.65, 1.97)	0.88 (0.50, 1.55)	0.851	
BMI (kg/m^2^)					0.722
BMI < 18.5	Ref.	0.56 (0.13, 2.43)	0.56 (0.12, 2.63)	0.442	
18.5 ≤ BMI < 24	Ref.	1.40 (0.82, 2.39)	0.79 (0.43, 1.43)	0.468	
24 ≤ BMI < 28	Ref.	0.78 (0.46, 1.33)	0.54 (0.30, 0.99)	0.045	
BMI ≥ 28	Ref.	1.32 (0.48, 3.61)	1.10 (0.35, 3.45)	0.832	
Marital status (%)					0.711
Married	Ref.	1.00 (0.69, 1.44)	0.67 (0.45, 1.01)	0.061	
Unmarried	Ref.	7.60 (0.39, 147.44)	1.55 (0.08, 31.32)	0.766	
Other	Ref.	1.63 (0.62, 4.31)	0.63 (0.16, 2.49)	0.745	
Education (%)					0.620
≤6 years	Ref.	1.01 (0.71, 1.44)	0.63 (0.42, 0.95)	0.038	
>7 years	Ref	2.42 (0.69, 8.42)	1.38 (0.39, 4.82)	0.442	
Residence status (%)					0.165
Rural	Ref.	1.39 (0.91, 2.13)	0.74 (0.47, 1.19)	0.240	
New-Urban	Ref.	0.65 (0.37, 1.17)	0.59 (0.30, 1.15)	0.093	
Smoking status (%)					0.694
Never smoked	Ref.	0.97 (0.65, 1.43)	0.65 (0.42, 1.01)	0.064	
Ever smoked	Ref.	1.38 (0.70, 2.73)	0.74 (0.34, 1.61)	0.481	
Alcohol-drinking status (%)					0.971
Non-drinker	Ref.	1.07 (0.73, 1.55)	0.65 (0.42, 1.00)	0.070	
Drinker	Ref.	1.05 (0.48, 2.30)	0.79 (0.35, 1.77)	0.562	
Tea-drinking status (%)					0.702
Non-tea drinker	Ref.	1.24 (0.74, 2.10)	0.73 (0.40, 1.34)	0.361	
Tea drinker	Ref.	0.93 (0.60, 1.46)	0.62 (0.38, 1.01)	0.063	
Dietary supplements use (%)					0.338
Yes	Ref.	2.22 (0.57, 8.58)	1.65 (0.39, 6.95)	0.555	
No	Ref.	0.99 (0.70, 1.41)	0.62 (0.42, 0.92)	0.024	

All analyses were adjusted for age, gender, BMI, marital status, education, residence status, smoking status, alcohol-drinking status, tea-drinking status and dietary supplement use.

## Data Availability

The raw survey data generated in this study are not publicly accessible due to restrictions related to protecting the privacy of human participants, as specified in our ethical approval. The de-identified minimal dataset is available from the corresponding author upon reasonable request and subject to a formal data sharing agreement.

## Supplementary Materials

The following supporting information can be downloaded at https://www.mdpi.com/article/10.3390/nu18172792/s1. Figure S1: Scree plot of eigenvalues from exploratory factor analysis of dietary patterns; Figure S2: Sensitivity analyses for the association between adherence to the livestock–poultry–seafood dietary pattern and incident suicidal ideation; Table S1: Baseline characteristics of participants included in the analytical sample and those excluded because of missing suicidal ideation data; Table S2: Comparison of the primary and inverse probability-weighted analyses of the association between dietary patterns and incident suicidal ideation; Table S3: Stratified analyses of associations between other dietary patterns and incident suicidal ideation; Table S4: The association of the livestock–poultry–seafood dietary pattern and depression and anxiety symptoms.

## Abbreviations

The following abbreviations are used in this manuscript:

NHANES	National Health and Nutrition Examination Survey
PCA	Principal component analysis
PHQ-9	9-Item Patient Health Questionnaire
GAD-7	7-Item Generalized Anxiety Disorder-7
BMI	Body mass index
ORs	Odds ratios
CIs	Confidence intervals
DI-GM	Dietary Index for Gut Microbiota
PUFAs	Polyunsaturated fatty acids
EPA	Eicosapentaenoic acid
DHA	Docosahexaenoic acid
