# Supplementary material for: Longitudinal Analysis of Associations Between Dietary Patterns and Incident Suicidal Ideation
Source: Nutrients. 2026 Aug 26;18(17):2792. doi: 10.3390/nu18172792 (PMC13567152; doi:10.3390/nu18172792)
Supplement: Supplementary file 1 [file nutrients-18-02792-s001.zip › Supplementary tables.pdf]

| Table S1. Baseline characteristics of participants included in the analytical sample and those excluded because of missing suicidal ideation data | | | |
|---|---|---|---|
| **Characteristics** | **Included** | **Excluded** | *P* |
| **N** | 7480 | 4076 | |
| **Age (years)** | 65.18 ± 14.23 | 55.53 ± 15.96 | <0.001 |
| **Sex** | | | 0.564 |
| Female | 3091 (41.32) | 2354 (57.75) | |
| Male | 4389 (58.68) | 1705 (41.83) | |
| Missing | - | 17 (0.42) | |
| **BMI (kg/m²)** | 24.64 ± 4.43 | 24.17 ± 4.19 | <0.001 |
| **Marital status (%)** | | | <0.001 |
| Married | 6184 (82.67) | 3416 (83.81) | |
| Unmarried | 216 (2.89) | 279 (6.85) | |
| Other | 1068 (14.28) | 372 (9.13） | |
| Missing | 12 (0.16) | 9 (0.22) | |
| **Education (%)** | | | <0.001 |
| ≤ 6 years | 6422 (85.86) | 1040 (25.52) | |
| > 7years | 979 (13.09) | 2954 (72.47) | |
| Missing | 79 (1.06) | 82 (2.01) | |
| **Residence status (%)** | | | <0.001 |
| Rural | 3670 (49.06) | 722 (17.71) | |
| New-Urban | 3718 (49.70) | 3139 (77.01) | |
| Urban | 55 (0.94) | 171 (4.20) | |
| Missing | 37 (0.49) | 44 (1.08) | |
| **Smoking status (%)** | | | <0.001 |
| Never smoked | 5946 (79.49) | 1676 (41.12) | |
| Ever smoked | 1534 (20.51) | 541 (13.27) | |
| Missing | - | 1859 (45.61) | |
| **Alcohol-drinking status (%)** | | | 0.020 |
| Non-drinker | 5892 (78.77) | 1694 (41.56) | |
| Drinker | 1588 (21.23) | 523 (12.83) | |
| Missing | - | 1859 (45.61) | |
| **Tea-drinking status(%)** | | | <0.001 |
| Non-tea drinker | 3275 (43.78) | 1370 (33.61) | |
| Tea drinker | 4175 (55.82) | 2657 (65.19) | |
| Missing | 30 (0.40) | 49 (1.20) | |
| **Dietary supplements use (%)** | | | <0.001 |

| | | | |
|---|---|---|---|
| Yes | 1485 (19.85) | 561 (13.76) | |
| No | 5963 (79.72) | 3474 (85.23) | |
| Missing | 32 (0.43) | 41 (1.01) | |
| **PHQ-9 (%)** | | | <0.001 |
| < 5 | 7322 (97.89) | 2019 (49.53) | |
| ≥ 5 | 158 (2.11) | 189 (4.64) | |
| Missing | - | 1868 (45.83) | |
| **GAD (%)** | | | <0.001 |
| < 10 | 7459 (99.72) | 2270 (55.69) | |
| ≥ 10 | 20 (0.27) | 29 (0.71) | |
| Missing | - | 1777 (43.60) | |

Table S2. Comparison of the primary and inverse probability-weighted analyses of the association between dietary patterns and incident suicidal ideation

| | **Included** | **Non-included** |
|---|---|---|
| Nut pattern | | |
| Q1 (-2.78, -0.77) | 1.00 (Ref.) | 1.00 (Ref.) |
| Q2 (-0.77, -0.36) | 1.16 (0.80, 1.68) | 0.93 (0.63, 1.37) |
| Q3 ( -0.36, 9.79) | 1.15 (0.79, 1.68) | 1.03 (0.71, 1.50) |
| Legume-grain-fungus pattern | | |
| Q1 (-3.79, -1.05) | 1.00 (Ref.) | 1.00 (Ref.) |
| Q2 (-1.05, -0.46) | 0.91 (0.63, 1.31) | 1.31 (0.91, 1.90) |
| Q3 (-0.46, 3.16) | 1.03 (0.72, 1.47) | 0.85 (0.57, 1.26) |
| **Livestock-poultry-seafood pattern** | | |
| Q1 (-3.17, -1.15) | 1.00 (Ref.) | 1.00 (Ref.) |
| Q2 (-1.15, -0.44) | 1.05 (0.75, 1.47) | 1.10 (0.77, 1.58) |
| Q3 (-0.44, 6.34) | **0.67 (0.45, 0.97)** | **0.68 (0.46, 1.00)** |
| Dairy pattern | | |
| Q1 (-2.69, -1.07) | 1.00 (Ref.) | 1.00 (Ref.) |
| Q2 (-1.07, -0.55) | 1.12 (0.79, 1.58) | 1.17 (0.81, 1.69) |
| Q3 (-0.55, 3.62) | 0.88 (0.61, 1.27) | 0.90 (0.61, 1.33) |
| High whole-grain-low egg pattern | | |
| Q1 (-3.63, -0.97) | 1.00 (Ref.) | 1.00 (Ref.) |
| Q2 (-0.97, -0.44) | 0.83 (0.56, 1.23) | 0.80 (0.53, 1.22) |

| | | |
|---|---|---|
| Q3 (-0.44, 4.52) | 1.17 (0.81, 1.69) | 1.18 (0.80, 1.73) |
| Vegetable-fruit pattern | | |
| Q1 (-8.43, -0.91) | 1.00 (Ref.) | 1.00 (Ref.) |
| Q2 (-0.91, -0.26) | 1.11 (0.79, 1.55) | 1.04 (0.73, 1.48) |
| Q3 (-0.26, 1.63) | 0.74 (0.50, 1.08) | 0.76 (0.50, 1.13) |
| Adjusted for sex, age and BMI, educational level, residence type, marital status, smoking, alcohol-drinking, tea-drinking status and dietary supplements use. | | |

Table S3 Stratified analyses of associations between other dietary patterns and incident suicidal ideation

| Characteristic | Q1 | Q2 | Q3 | *P* for trend | *P* for interaction |
|---|---|---|---|---|---|
| **Nut pattern** | | | | | |
| Age (years) | | | | | 0.356 |
| Age < 60 | Ref. | 0.61 (0.27, 1.41) | 0.80 (0.38, 1.68) | 0.732 | |
| Age ≥ 60 | Ref. | 1.30 (0.86, 1.96) | 0.55 (0.77, 1.83) | 0.437 | |
| Sex (%) | | | | | 0.403 |
| Male | Ref. | 1.32 (0.82, 2.14) | 1.08 (0.65, 1.79) | 0.811 | |
| Female | Ref. | 0.93 (0.52, 1.67) | 1.23 (0.70, 2.16) | 0.445 | |
| BMI (kg/m²) | | | | | 0.136 |
| BMI < 18.5 | Ref. | 0.35 (0.07, 1.82) | 0.31 (0.06, 1.49) | 0.111 | |
| 18.5 ≤ BMI < 24 | Ref. | 1.69 (0.90, 3.18) | 2.05 (1.10, 3.80) | 0.025 | |
| 24 ≤ BMI < 28 | Ref. | 1.16 (0.65, 2.07) | 1.12 (0.62, 2.05) | 0.714 | |
| BMI ≥ 28 | Ref. | 0.99 (0.36, 2.76) | 0.42 (0.14, 1.33) | 0.134 | |
| Education (%) | | | | | 0.241 |
| ≤ 6 years | Ref. | 1.27 (0.86, 1.88) | 1.19 (0.79, 1.78) | 0.427 | |
| > 7years | Ref | 0.47 (0.13, 1.69) | 0.98 (0.35, 2.72) | 0.882 | |
| Dietary supplements use (%) | | | | | 0.348 |
| Yes | Ref. | 0.53 (0.13, 2.09) | 1.12 (0.37, 3.43) | 0.865 | |
| No | Ref. | 1.24 (0.84, 1.84) | 1.18 (0.79, 1.76) | 0.445 | |
| **Legume-grain-fungus pattern** | | | | | |
| Age (years) | | | | | 0.092 |
| Age < 60 | Ref. | 1.97 (0.80, 4.83) | 2.27 (0.95, 5.45) | 0.075 | |
| Age ≥ 60 | Ref. | 0.76 (0.50, 1.15) | 0.87 (0.58, 1.30) | 0.485 | |
| Sex (%) | | | | | 0.694 |
| Male | Ref. | 1.02 (0.62, 1.67) | 1.22 (0.76, 1.97) | 0.398 | |
| Female | Ref. | 0.81 (0.47, 1.41) | 0.84 (0.49, 1.45) | 0.530 | |
| BMI (kg/m²) | | | | | 0.529 |
| BMI < 18.5 | Ref. | 2.45 (0.53, 11.41) | 1.32 (0.24, 7.12) | 0.806 | |
| 18.5 ≤ BMI < 24 | Ref. | 0.64 (0.35, 1.16) | 0.99 (0.58, 1.68) | 0.976 | |

| | | | | | |
|---|---|---|---|---|---|
| 24 ≤ BMI < 28 | Ref. | 0.90 (0.50, 1.59) | 0.97 (0.55, 1.71) | 0.915 | |
| BMI ≥ 28 | Ref. | 1.79 (0.57, 5.67) | 1.26 (0.40, 3.97) | 0.789 | |
| Education (%) | | | | | 0.470 |
| ≤ 6 years | Ref. | 0.98 (0.66, 1.45) | 1.05 (0.72, 1.54) | 0.787 | |
| > 7years | Ref | 0.43 (0.14, 1.38) | 0.71 (0.26, 1.92) | 0.564 | |
| Dietary supplements use (%) | | | | | 0.665 |
| Yes | Ref. | 0.69 (0.18, 2.65) | 1.39 (0.44, 4.42) | 0.522 | |
| No | Ref. | 0.92 (0.63, 1.36) | 0.99 (0.68, 1.44) | 0.965 | |
| **Dairy pattern** | | | | | |
| Age (years) | | | | | 0.475 |
| Age < 60 | Ref. | 1.06 (0.51, 2.21) | 0.70 (0.32, 1.54) | 0.344 | |
| Age ≥ 60 | Ref. | 0.13 (-0.27, 0.52) | -0.01 (-0.43, 0.41) | 0.993 | |
| Sex (%) | | | | | 0.662 |
| Male | Ref. | 1.06 (0.67, 1.70) | 0.94 (0.58, 1.52) | 0.798 | |
| Female | Ref. | 1.14 (0.77, 1.69) | 0.99 (0.65, 1.50) | 0.359 | |
| BMI (kg/m²) | | | | | 0.957 |
| BMI < 18.5 | Ref. | 0.80 (0.17, 3.68) | 0.76 (0.18, 3.23) | 0.721 | |
| 18.5 ≤ BMI < 24 | Ref. | 1.30 (0.76, 2.22) | 0.79 (0.43, 1.44) | 0.458 | |
| 24 ≤ BMI < 28 | Ref. | 0.98 (0.56, 1.70) | 0.94 (0.53, 1.66) | 0.827 | |
| BMI ≥ 28 | Ref. | 0.95 (0.35, 2.58) | 0.73 (0.25, 2.19) | 0.590 | |
| Education (%) | | | | | 0.784 |
| ≤ 6 years | Ref. | 1.07 (0.74, 1.54) | 0.87 (0.59, 1.28) | 0.509 | |
| > 7years | Ref | 1.94 (0.57, 6.65) | 1.29 (0.38, 4.45) | 0.847 | |
| Dietary supplements use (%) | | | | | 0.194 |
| Yes | Ref. | 0.70 (0.17, 2.92) | 1.65 (0.39, 6.95) | 0.240 | |
| No | Ref. | 0.99 (0.70, 1.41) | 0.62 (0.42, 0.92) | 0.310 | |
| **High whole-grain-low egg pattern** | | | | | |
| Age (years) | | | | | 0.850 |
| Age < 60 | Ref. | 0.71 (0.31, 1.65) | 0.93 (0.43, 2.00) | 0.976 | |
| Age ≥ 60 | Ref. | 0.84 (0.54, 1.32) | 1.25 (0.83, 1.90) | 0.257 | |
| Sex (%) | | | | | 0.838 |
| Male | Ref. | 0.78 (0.47, 1.30) | 1.06 (0.65, 1.71) | 0.727 | |
| Female | Ref. | 0.89 (0.48, 1.65) | 1.31 (0.74, 2.32) | 0.293 | |
| BMI (kg/m²) | | | | | 0.007 |
| BMI < 18.5 | Ref. | 0.14 (0.01, 1.31) | 0.83 (0.21, 3.33) | 0.776 | |
| 18.5 ≤ BMI < 24 | Ref. | 0.61 (0.34, 1.09) | 0.69 (0.40, 1.20) | 0.202 | |
| 24 ≤ BMI < 28 | Ref. | 1.65 (0.87, 3.13) | 1.98 (1.05, 3.74) | 0.038 | |
| BMI ≥ 28 | Ref. | 0.61 (0.13, 2.80) | 2.49 (0.78, 7.97) | 0.052 | |
| Education (%) | | | | | 0.993 |
| ≤ 6 years | Ref. | 0.83 (0.55, 1.26) | 1.16 (0.79, 1.71) | 0.382 | |
| > 7years | Ref | 0.77 (0.22, 2.62) | 1.16 (0.38, 3.53) | 0.674 | |
| Dietary supplements use (%) | | | | | 0.535 |
| Yes | Ref. | 0.69 (0.22, 2.19) | 0.58 (0.16, 2.16) | 0.405 | |
| No | Ref. | 0.84 (0.56, 1.28) | 1.23 (0.84, 1.81) | 0.210 | |
| **Vegetable-fruit pattern** | | | | | |
| Age (years) | | | | | 0.340 |
| Age < 60 | Ref. | 0.99 (0.48, 2.06) | 1.11 (0.53, 2.36) | 0.777 | |
| Age ≥ 60 | Ref. | 1.10 (0.75, 1.60) | 0.61 (0.39, 0.97) | 0.056 | |
| Sex (%) | | | | | 0.436 |
| Male | Ref. | 0.91 (0.58, 1.42) | 0.67 (0.41, 1.10) | 0.120 | |

| | | | | | |
|---|---|---|---|---|---|
| Female | Ref. | 1.40 (0.83, 2.34) | 0.84 (0.46, 1.56) | 0.717 | |
| BMI (kg/m²) | | | | | 0.550 |
| BMI < 18.5 | Ref. | 0.50 (0.09, 2.78) | 1.19 (0.31, 4.59) | 0.848 | |
| 18.5 ≤ BMI < 24 | Ref. | 0.93 (0.55, 1.57) | 0.68 (0.38, 1.24) | 0.224 | |
| 24 ≤ BMI < 28 | Ref. | 1.18 (0.70, 2.01) | 0.60 (0.32, 1.14) | 0.152 | |
| BMI ≥ 28 | Ref. | 1.99 (0.68, 5.78) | 1.30 (0.39, 4.39) | 0.668 | |
| Education (%) | | | | | 0.409 |
| ≤ 6 years | Ref. | 1.14 (0.79, 1.62) | 0.82 (0.54, 1.23) | 0.404 | |
| > 7years | Ref | 0.87 (0.33, 2.28) | 0.38 (0.11, 1.28) | 0.124 | |
| Dietary supplements use (%) | | | | | 0.035 |
| Yes | Ref. | 0.56 (0.13, 2.43) | 1.70 (0.54, 5.34) | 0.292 | |
| No | Ref. | 1.16 (0.82, 1.64) | 0.65 (0.43, 0.99) | 0.075 | |

All analyses were adjusted for age, gender, BMI, marital status, education, residence status, smoking status, alcohol-drinking status, tea-drinking status and dietary supplement use.

Table S4 The association of the livestock-poultry-seafood dietary pattern and depression and anxiety symptoms

| Livestock-poultry-seafood dietary pattern | PHQ-9 | | GAD | |
|---|---|---|---|---|
| | OR (95% CIs) | *P* | OR (95% CIs) | *P* |
| Q1 | Ref. | | Ref. | |
| Q2 | -0.12 (-0.18, -0.05) | <0.001 | -0.18 (-0.26, -0.09) | <0.001 |
| Q3 | -0.08 (-0.14, -0.02) | 0.012 | -0.06 (-0.15, 0.02) | 0.129 |
| *P* for trend | 0.011 | | 0.115 | |
